# An abrupt loss of capture during permanent His‐bundle pacing: Assessment of mechanism underlying late capture threshold rise

**DOI:** 10.1002/joa3.13165

**Published:** 2024-10-18

**Authors:** Hiroyuki Kato, Satoshi Yanagisawa, Ryusuke Ota, Yasuya Inden, Toyoaki Murohara

**Affiliations:** ^1^ Department of Cardiology Japan Community Health Care Organization Chukyo Hospital Nagoya Japan; ^2^ Department of Cardiology Nagoya University Graduate School of Medicine Nagoya Japan

**Keywords:** capture threshold, conduction system pacing, fibrosis, His‐bundle pacing, left bundle branch area pacing

## Abstract

A His‐bundle (HB) capture threshold rise is still a significant concern in permanent His‐bundle pacing (HBP). We present a case where an abrupt increase in HB threshold and loss of capture occurred even after 3.5 years of stable permanent HBP for an atrioventricular block. The development of local fibrosis around the lead helix and the change in an insertion angle of the lead might adversely affect the HB capture threshold in the chronic phase.

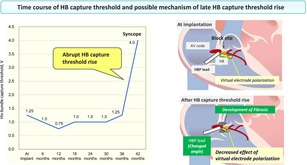

A 79‐year‐old man who had an intermittent second‐degree atrioventricular block and mildly reduced left ventricular ejection fraction (LVEF) of 48% was admitted to Chukyo Hospital for pacemaker implantation. He had no history of structural heart disease. Before pacemaker implantation, neither myocardial wall thinning, aneurysms, nor late gadolinium enhancement was confirmed on cardiac magnetic resonance imaging. A His‐bundle pacing (HBP) lead (SelectSecure™ model 3830; Medtronic) implantation was selected to prevent further development of heart failure. During the implantation, a split His‐bundle (HB) potential was confirmed on unipolar electrograms of the lead using a specialized sheath (C315HIS; Medtronic). The second‐degree type II intra‐Hisian block was identified based on the absence of the second component of the split HB potential not followed by a ventricular electrogram (Figure 1A). After fixation of the lead, a selective HB capture was successfully achieved without an exit block by high‐rate pacing of 140 ppm. The sheath was gently removed, leaving a sufficient lead slack. The capture thresholds of HB and local ventricle at implant were 1.25 V and 1.50 V at 1.0 ms, respectively. R‐wave amplitude was 1.6 mV. After the implantation, the HB capture threshold showed no deterioration during the follow‐up period, and a pacing output was fixed to 3.0 V at 1.0 ms (Figure 2). After 36 months, the local ventricular capture threshold gradually increased to 2.5 V at 1.0 ms, whereas the HB capture threshold remained stable. No alerts were transmitted in remote monitoring during the follow‐up. The LVEF improved to 55% after 1 year.

**FIGURE 1:**
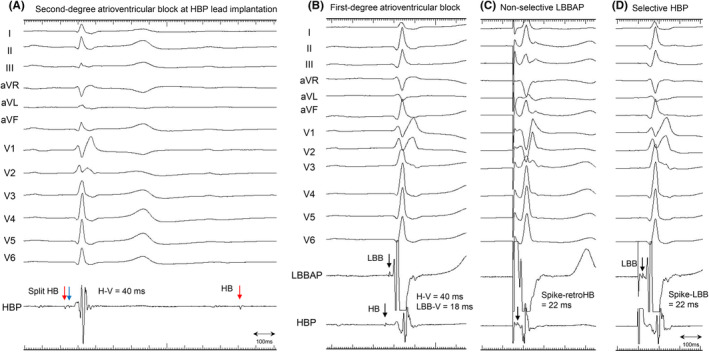
Twelve‐lead electrocardiogram and unipolar electrograms on HBP and LBBAP lead tip. (A) Second‐degree type II intra‐Hisian block at HBP implantation. (First beat) the split HB potential (*red and blue arrows*) was identified with an H‐V interval of 40 ms during intrinsic conduction. (Second beat) the second component of the split HB potential (*blue arrow*) disappeared, resulting in the intra‐Hisian block. (B) First‐degree atrioventricular block at lead revision. After additional LBBAP lead deployment into the interventricular septum, the LBB potential was recorded. An H‐V interval and an LBB‐V interval were 40 ms and 18 ms, respectively. (C) During the non‐selective LBBAP at the pacing output of 5.0 V at 0.4 ms, the retrograde HB potential was identified on the HBP lead, showing that the interval from the pacing spike to retrograde HB potential was 22 ms. Note that right bundle branch block was corrected, and R‐wave amplitude in lead I was higher than that during intrinsic conduction; these findings were possibly due to the capture of the fasciculoventricular pathway in the muscular septum by high‐output pacing.^1^ (D) During the selective HBP at the pacing output of 4.0 V at 1.0 ms, the anterograde LBB potential was identified on the LBBAP lead, showing that the interval from the pacing spike to the LBB potential was 22 ms, which was identical to the interval of the retrograde conduction presented in (C). HB, His‐bundle; HBP, His‐bundle pacing; H‐V, His‐ventricle; LBB, left bundle branch; LBBAP, left bundle branch area pacing; LBB‐V, left bundle branch‐ventricle.

**FIGURE 2:**
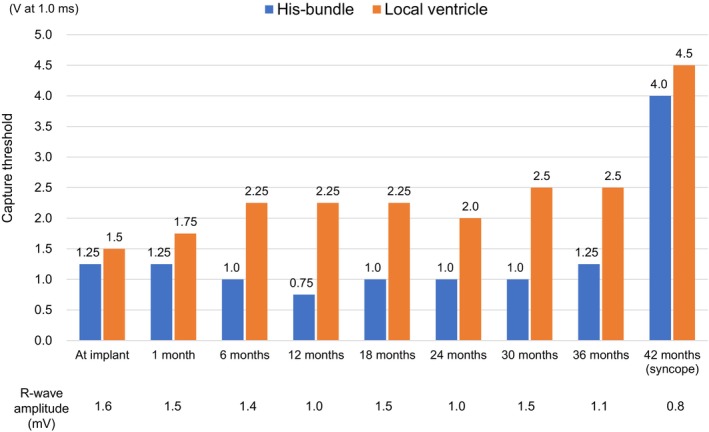
Changes in the His‐bundle and local ventricular capture thresholds after HBP implantation. The pacing thresholds were examined in a sitting position at each outpatient visit. The His‐bundle capture threshold was maintained stable for 3 years, whereas the local ventricular capture threshold gradually increased. HBP, His‐bundle pacing.

Forty‐two months after the implantation, the patient came to our emergency department with a sudden loss of consciousness. Both the HB and local ventricular capture thresholds significantly rose (4.0 V and 4.5 V at 1.0 ms, respectively) with a loss of capture on the device interrogation. The thresholds did not change considerably between unipolar and bipolar pacing configurations. Although a chest X‐ray image showed no apparent lead dislodgement, a slightly decreased lead slack was observed compared to the time of initial implantation (Figure 3A,B). The patient was scheduled for the HBP lead extraction and new lead implantation of left bundle branch area pacing (LBBAP).

**FIGURE 3:**
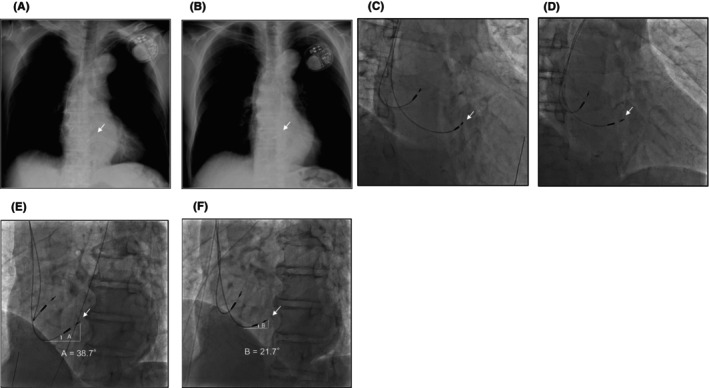
X‐rays and fluoroscopic images. (A) Chest X‐ray at implantation. (B) Chest X‐ray after the threshold rise. (C) Fluoroscopic image in the RAO 30° at implantation. (D) Fluoroscopic image in the RAO 30° at lead revision. (E) Fluoroscopic image in the LAO 50° at implantation. The lead insertion angle (angle A) was 38.7°. (F) Fluoroscopic image in the LAO 50° at lead revision. The lead insertion angle (angle B) was 21.7°, smaller than angle A due to the decreased lead slack. Images A and B were taken at the standing position, and images C–F were taken at the supine position. LAO, left anterior oblique; RAO, right anterior oblique.

An unchanged H‐V interval of 40 ms compared to the initial implantation was observed during intrinsic atrioventricular conduction (Figure 1B). The fluoroscopic images demonstrated that the HBP lead was pulled toward the right atrium due to a migration of the lead slack into the left brachiocephalic vein, resulting in a more horizontal insertion angle of the lead than at the initial implantation despite the same supine position (Figure 3C–F, Videos [Link], [Link], [Link]). Firstly, a successful LBBAP was achieved using a 3830 lead and a C315HIS sheath.^2^ The left bundle branch (LBB) potential was confirmed with an LBB‐QRS interval of 18 ms (Figure 1B). The LBB could be captured with the R‐wave peak time of 65 ms in lead V6 at the pacing output of 1.5 V at 0.4 ms. The interval from the LBBAP spike to the retrograde HB potential was 22 ms, which was identical to the interval from the HBP spike to the LBB potential, suggesting maintenance of bidirectional infra‐Hisian conduction (Figure 1C,D). After the LBBAP lead implantation, the HBP lead was extracted only by manual traction. When the HBP lead was manually pulled counterclockwise with a struggle for approximately 3 min, the lead tip was successfully removed. A gross inspection of the extracted lead showed that fibrous tissue was stuck around the lead helix (Figure 4). The patient had an excellent clinical course with stable LBBAP parameters and preserved LVEF after 1 year.

**FIGURE 4:**
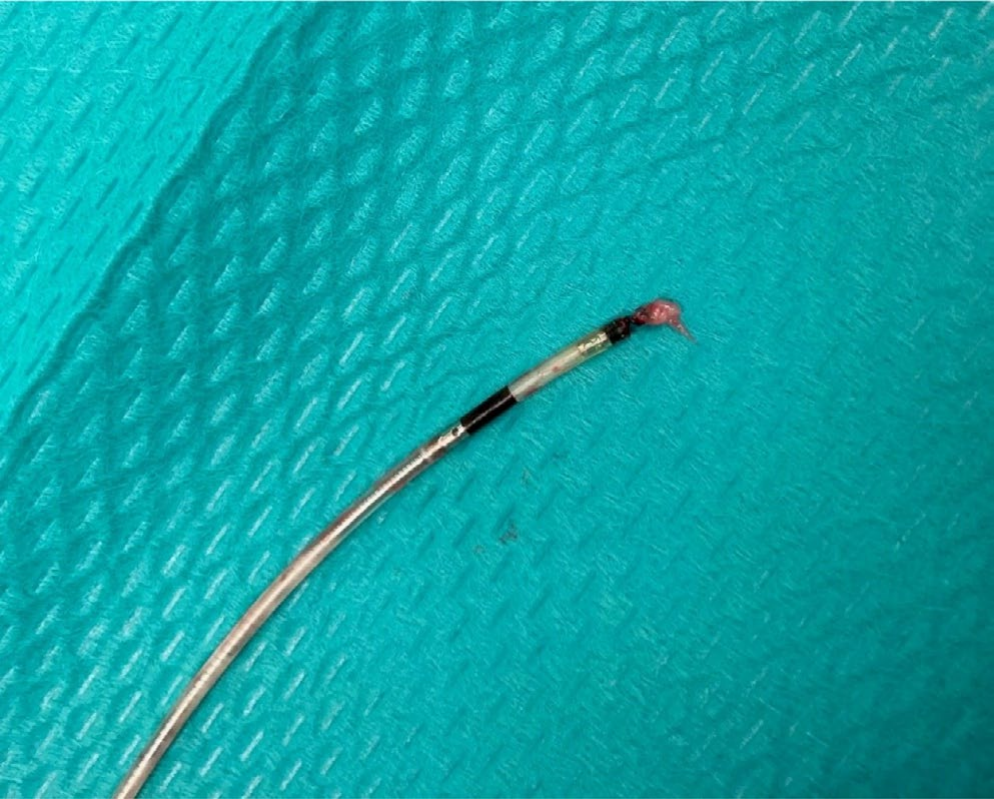
A gross inspection of the extracted HBP lead. HBP, His‐bundle pacing.

A rise in the HB capture threshold is a significant concern when performing permanent HBP. An HB capture threshold rise generally occurs in an early phase, likely resulting from procedural‐related factors.^3^ Meanwhile, a late HB capture threshold rise has been reported in approximately one‐third of the patients during the long‐term follow‐up period.^3^, ^4^ Although the exact mechanism underlying the late threshold rise remains unclear, previous studies have speculated that various mechanisms caused an HB capture threshold rise in a chronic phase: local fibrosis leading to exit block, progression of underlying conduction disease, and a micro‐dislodgement of an HBP lead caused by tricuspid valve motion.^3^ In the present case, the abrupt loss of capture occurred even though the HB capture threshold remained stable for 3 years. Moreover, the HBP lead helix had been firmly fixed to the HB region, suggesting that a micro‐dislodgement of the lead was unlikely. Therefore, the progression of local fibrosis around the lead helix was one possible explanation for the late threshold rise.

We further speculated that the decreased insertion angle of the lead was another mechanism underlying the abrupt threshold rise in this case. Virtual electrode polarization is an electrical phenomenon typically demonstrated in pacing stimulation on anisotropic cardiac tissue.^4^ Previous studies reported that the virtual electrode polarization by HBP might play an essential role in promoting propagation in diseased tissue around the HB area.^4^, ^5^ In the present case with the preexisting intra‐Hisian block and the development of local fibrosis around the lead helix, the change in the insertion angle of the lead might electrically influence the facing attached tissue with the changes in orientation and direction of the fibers, possibly resulting in the reduced virtual electrode effect and subsequent abrupt capture threshold rise with an exit block.

There were several limitations to elucidate the mechanism for the abrupt threshold rise in the present case. The first was the lack of regular follow‐up evaluation on X‐rays and fluoroscopic images; therefore, it was unclear whether the decreased insertion angle of the lead occurred gradually or abruptly. The second was the lack of a microscopic observation of tissue around the helix. In addition, the assessment of cardiomyopathy using cardiac magnetic resonance imaging was performed before pacemaker implantation only but not after the abrupt threshold increase. Further studies will be required to determine the factors related to the HB capture threshold rise in the chronic phase, including the abrupt rise.

## FUNDING INFORMATION

None.

## CONFLICT OF INTEREST STATEMENT

Dr. Yanagisawa is affiliated with a department sponsored by Medtronic, Japan. Other authors have no conflicts of interest.

## ETHICS APPROVAL STATEMENT

This study was approved by the ethics committee of Chukyo Hospital. The study was performed in accordance with the principles of the Declaration of Helsinki.

## PATIENT CONSENT STATEMENT

The authors confirm that written consent for submission and publication of this case report, including images and associated text, has been obtained from the patient.

## CLINICAL TRIAL REGISTRATION

N/A.

## REGISTRY AND REGISTRATION NO. OF THE STUDY/TRIAL

N/A.

## ANIMAL STUDIES

N/A.

## Supporting information


**Video S1.** Fluoroscopic image in posterior–anterior view at the HBP lead implantation.

**Video S2.** Fluoroscopic image after the HB capture threshold rise.

**Video S3.** Fluoroscopic image after the HB capture threshold rise.

## Data Availability

The datasets generated and/or analyzed during the current study are available from the corresponding author upon reasonable request.
